# Primary High-Grade Spinal Sarcomas: A Retrospective Review of 10 Cases

**DOI:** 10.7759/cureus.108569

**Published:** 2026-05-09

**Authors:** Ryunosuke Fukushi, Makoto Emori, Hiroyuki Tsuchie, Naoya Nakahashi, Junya Shimizu, Yasutaka Murahashi, Naohisa Miyakoshi, Atsushi Teramoto

**Affiliations:** 1 Orthopedic Surgery, Sapporo Medical University School of Medicine, Sapporo, JPN; 2 Orthopedic Surgery, Akita University Graduate School of Medicine, Akita, JPN

**Keywords:** activities of daily living, primary spinal sarcoma, spinal reconstruction, spine, surgical resection

## Abstract

Introduction: Aggressive surgical resection with adequate margins is associated with improved local control and survival in selected patients with primary spinal sarcomas. However, anatomical constraints often preclude complete resection, and outcomes for unresectable tumors remain poor. This retrospective cohort study aimed to compare oncological and functional outcomes between patients with primary spinal sarcomas who underwent surgical resection with reconstruction and those who were managed without resection.

Methods: Ten patients with primary spinal sarcomas treated between 2010 and 2020 were retrospectively reviewed. Six patients underwent surgical resection with spinal reconstruction (R group), and four were treated without resection (N group). The mean age was 51.8 years (range, 15-71 years) in the R group and 73.0 years (range, 53-91 years) in the N group. Clinical variables, recurrence, metastasis, overall survival (OS), and activities of daily living (ADL), including ambulatory status, were evaluated. OS was analyzed using the Kaplan-Meier method and compared using the log-rank test.

Results: Tumor size at initial presentation was comparable between the groups (48.3 mm (range, 24 mm-68 mm) in the R group and 48.8 mm (range, 38mm-75 mm) in the N group). Metastases occurred in three patients (50%) in the R group and three patients (75%) in the N group. Two patients (33%) in the R group remained disease-free, whereas all patients in the N group died during follow-up. The five-year OS rate was 60% in the R group and 0% in the N group, with no significant difference (p=0.23). Ambulatory function was preserved in five patients (83%) in the R group and one patient (25%) in the N group.

Conclusions: Although a definitive survival benefit could not be demonstrated, surgical resection with reconstruction was associated with superior postoperative functional outcomes, suggesting a role in preserving ambulation and quality of life in patients with primary spinal sarcomas.

## Introduction

Primary sarcoma of the spine is an exceedingly rare malignancy, representing less than 5% of all primary musculoskeletal sarcomas [1]. Its rarity poses a fundamental challenge to the establishment of standardized treatment strategies. The complex anatomy of the spine, its proximity to the spinal cord and cauda equina, and the limited surgical margins inherent to the vertebral column make curative resection extremely challenging. Despite advances in spinal oncology surgery, achieving safe and wide en bloc margins, the gold standard for high-grade sarcomas of the extremities, remains substantially more difficult in the axial skeleton [2].

Owing to these anatomical constraints, surgical plans are often individualized. The ability to perform complete resection varies widely depending on tumor location, invasion of neural elements, patient age, comorbidities, and histological subtype. Consequently, a therapeutic algorithm comparable to that used for sarcomas of the extremities is lacking. Furthermore, because the disease is extremely uncommon, most publications consist of small retrospective series or pooled analyses, and robust prognostic factors or outcome predictors remain insufficiently defined.

Despite these limitations, the available literature strongly suggests that aggressive surgical resection combined with appropriate oncologic therapy is associated with improved local control and overall survival (OS) in selected patients with primary high-grade spinal sarcoma [3-7]. However, when complete resection is not feasible, treatment outcomes remain poor, highlighting the urgent need for improved management strategies and more comprehensive data.

In this study, we reviewed 10 consecutive patients with primary spinal sarcoma treated at our institution over a decade. We compared clinical outcomes, including survival, recurrence, metastasis, and functional prognosis, between patients who underwent surgical resection with reconstruction (R group) and those in whom resection was not possible (N group). By analyzing these two groups, we sought to clarify the impact of surgical intervention on overall disease behavior and functional recovery.

## Materials and methods

Ethics statement

We conducted this study in compliance with the principles of the Declaration of Helsinki. The study protocol was reviewed and approved by the Institutional Review Board of the institution where the surgeries and measurements were performed (approval code: 362-6).

Study design and settings

This retrospective study reviewed 10 consecutive patients diagnosed with primary spinal sarcoma and treated at our institution over a 10-year period (2010-2020).

Inclusion and exclusion criteria

Because primary malignant sarcomas of the spine are exceptionally rare and heterogeneous in biological behavior, all cases of histologically confirmed primary sarcomas originating from the spinal vertebrae or sacrum were included. Metastatic spinal tumors, hematologic malignancies, and tumors arising from paraspinal soft tissues without bony involvement were excluded to ensure diagnostic homogeneity.

Participants

The cohort was subdivided into two groups based on whether surgical resection with spinal reconstruction was performed. The resection group (R group) consisted of six patients (three men and three women), whereas the non-resection group (N group) comprised four patients (one man and three women). The mean age in the R group was 51.8 years (range, 15-71 years), compared with 73.0 years (range, 53-91 years) in the N group, reflecting that older patients or those with greater comorbidities tended to be considered unsuitable for extensive surgical intervention. Histopathological diagnoses demonstrated marked diversity, reflecting the heterogeneous nature of axial sarcomas. Treatment allocation was not randomized because this was a retrospective study. Decisions regarding surgical resection were made through multidisciplinary discussion, taking into account tumor location, extent of invasion, histological subtype, patient age, comorbidities, and the feasibility of achieving safe surgical margins. Imaging findings, including MRI and CT, as well as histopathological diagnosis obtained through biopsy, were essential in determining resectability and guiding treatment strategy.

In the R group, tumors included Ewing sarcoma (Th6, S2) in two patients, leiomyosarcoma (L4) in two patients, chondrosarcoma (C6) in one patient, and undifferentiated pleomorphic sarcoma (Th10) in one patient. In contrast, the N group consisted of two cases of osteosarcoma (Th9 and Th11/12) and one case each of rhabdomyosarcoma (L5) and undifferentiated pleomorphic sarcoma (L4). These histological differences partly reflect intrinsic differences in resectability, as osteosarcoma and other aggressive subtypes often demonstrate extensive local invasion at diagnosis, precluding the achievement of safe surgical margins.

Variables

Radiological evaluations included plain radiography, computed tomography, and magnetic resonance imaging for all patients. Tumor extension and anatomical involvement were assessed using the Weinstein-Boriani-Biagini (WBB) classification, which enabled systematic evaluation of transverse and longitudinal tumor spread and guided surgical decision-making [2]. All cases were discussed at a multidisciplinary conference involving orthopedic oncologists, spine surgeons, radiologists, and medical oncologists to determine resectability and the need for adjunctive therapy. Clinical parameters evaluated included baseline demographics, tumor location, histology, WBB classification, details of surgical resection and reconstruction (if performed), adjuvant chemotherapy or radiotherapy, postoperative complications, and oncologic outcomes. Functional outcomes were assessed using activities of daily living (ADL), with specific emphasis on postoperative ambulatory status, a critical determinant of quality of life in patients with spinal sarcoma. OS was calculated from the date of diagnosis to the date of death or last follow-up.

Statistical analysis

OS curves were generated using the Kaplan-Meier method, and differences between the R and N groups were analyzed using the log-rank test. Statistical significance was defined as p<0.05. Due to the small number of cases inherent to rare spinal sarcomas, the analysis was primarily descriptive but aimed to identify clinically meaningful trends regarding the impact of resection on survival and functional outcomes.

## Results

A total of 10 patients with primary spinal sarcoma were included, with six patients undergoing surgical resection and reconstruction (R group) and four treated without resection (N group). Tumor size at the initial visit was comparable between the groups, with a mean diameter of 48.3 mm (range, 24 mm-68 mm) in the R group and 48.8 mm (range, 38 mm-75 mm) in the N group. According to the WBB classification [2], tumors in the R group were categorized as IIA in one case and IIB in five cases, whereas those in the N group included IIA in two cases and IIB in two cases, indicating that the majority of tumors in both groups had breached the vertebral body cortex and extended into the paravertebral region. Adjuvant therapy varied across the groups. In the R group, four patients received chemotherapy, two received radiation therapy, and one underwent carbon-ion radiotherapy, reflecting an aggressive multimodal treatment approach. In the N group, three patients received chemotherapy and two received radiation therapy, emphasizing reliance on nonsurgical strategies when en bloc resection was not feasible.

Metastasis to distant organs occurred in six patients (60%). Metastatic disease developed in three patients in the R group (50%) and three patients in the N group (75%), suggesting that resectable tumors may exhibit slower biological progression than unresectable tumors. Tumor recurrence was observed in two patients (33%) in the R group. Local control could not be evaluated in the N group because no en bloc resection was attempted.

Oncologic outcomes differed markedly between the groups. In the R group, two patients (33%) achieved continuous disease-free (CDF) status, one (17%) remained alive with disease (AWD), two (33%) died of disease (DOD), and one (17%) died of other causes (DOC). In contrast, all patients in the N group had died by the final follow-up, including three DOD cases (75%) and one DOC case (25%) (Table 1). The mean follow-up duration was 77 months in the resection group and 14.5 months in the non-resection group, the latter corresponding to OS because all patients in the non-resection group died during the follow-up period.

**Table 1:** Patient demographics, tumor characteristics, treatment modalities, and clinical outcomes This table summarizes each patient’s background, including age, sex, tumor histology, spinal level, initial tumor size, treatment strategy (resection with reconstruction vs. non-resection), adjuvant therapies, postoperative complications, recurrence, metastasis, and final oncological outcome. AWD: alive with disease; ADL: activities of daily living; OS: overall survival; CDF: continuous disease-free

Patient background			
		Group R	Group N
Number of patients		6	4
Sex (men:women)		3:3	1:3
Age (years)		51.8	73
Tumor type	Ewing sarcoma	2	
	Leiomyosarcoma	2	
	Chondrosarcoma	1	
	Undifferentiated pleomorphic sarcoma	1	1
	Osteosarcoma		2
	Rhabdomyosarcoma		1
Location of tumor	Cervical	1	0
	Thoracic	2	2
	Lumbar	2	2
	Sacrum	1	0
Tumor diameter at initial diagnosis (cm)		48.3	48.8
Weinstein classification	ⅡA	1	2
	ⅡB	5	2
Treatment plan			
Chemotherapy	+	4	3
	-	2	1
Radiation therapy	+	2	2
	-	4	2
Carbon ion radiotherapy	+	1	0
	-	5	4
Proton therapy	+	0	0
	-	6	4
Consequence			
Recurrence	+	2	-
	-	4	-
Metastasis to other organs	+	3	3
	-	3	1
Survival time (month）		69.8	14.5
Disease-free survival (month）		39.8	-
5-year OS(%)		60	0
Outcome	CDF	2	0
	AWD	1	0
	Dead	3	4
Cause of death	Dead by disease	2	3
	Death due to other cause	1	1
ADL	Walkable	5	1
	Unable to walk	1	3

**Figure 1:**
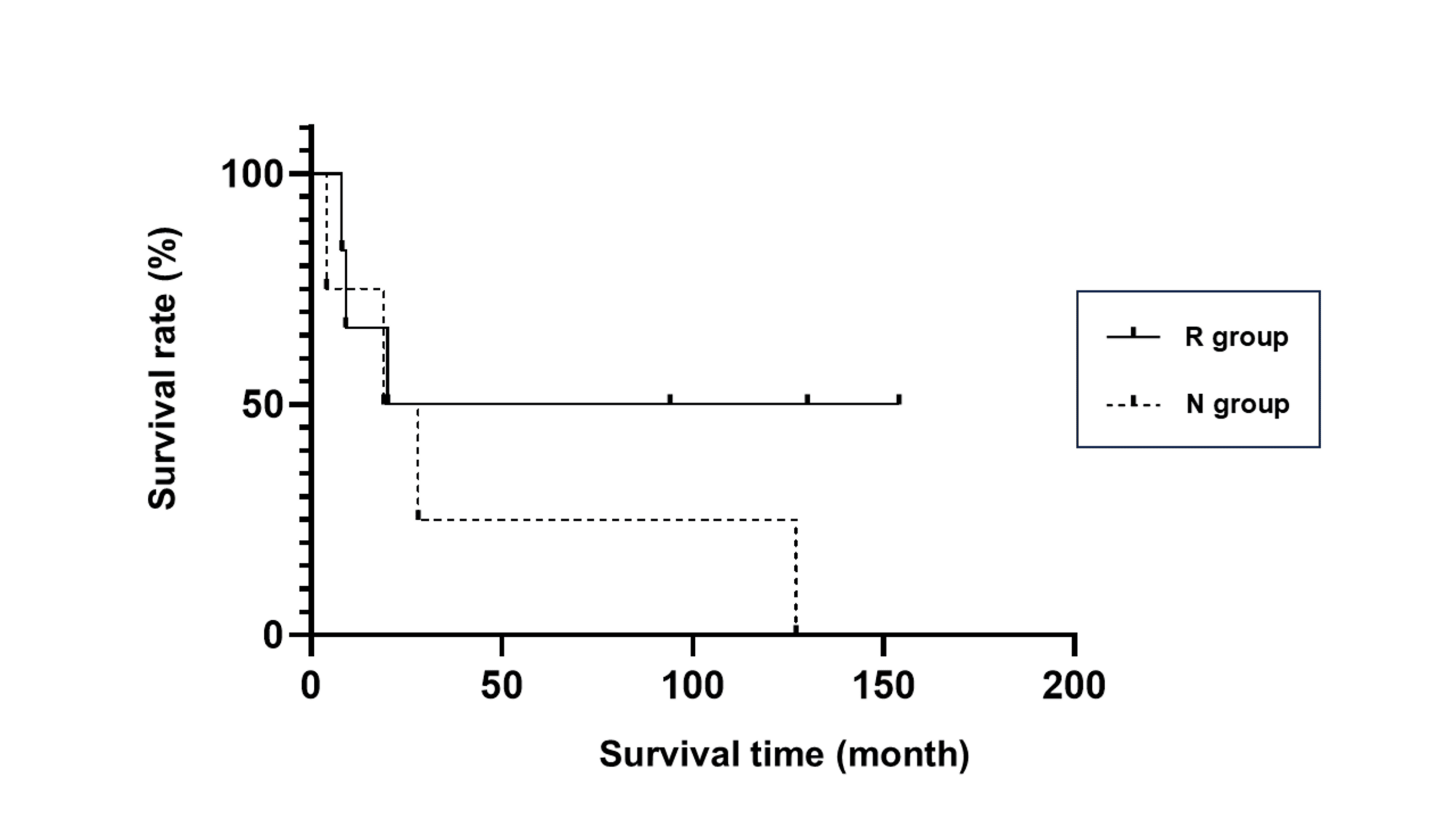
Kaplan-Meier analysis of OS in patients with primary high-grade spinal sarcoma OS was compared between the resection with reconstruction group (R group) and the non-resected group (N group), demonstrating a trend toward improved survival in the R group. OS: overall survival

Kaplan-Meier analysis demonstrated numerically higher OS in the resection group. The five-year OS rate was 60% in the R group and 0% in the N group; however, this difference was not statistically significant, likely due to the limited sample size (log-rank test, p=0.23) (Figure 1).

Functional outcomes also differed between the two groups. Five of the six patients (83%) in the R group remained ambulatory at the final follow-up, whereas only one of the four patients (25%) in the N group retained independent ambulation (Table 1). Individual concomitant oncologic treatments and final outcomes for each patient are summarized in Table 2.

**Table 2:** Patient-level details of concomitant oncological treatments and final outcomes This table presents individual patient data, including surgical status (resection with reconstruction or non-resection), concomitant oncologic therapies (chemotherapy, radiation therapy, carbon-ion radiotherapy, and proton therapy), treatment combinations, and final oncologic outcomes at the last follow-up (disease-free, AWD, or dead). The table allows direct comparison of treatment modalities and outcomes on a per-patient basis. (+) indicates that treatment was administered; (-) indicates that no treatment was administered. AWD: alive with disease; CDF: continuous disease-free

		Chemotherapy	Radiation therapy	Carbon ion radiotherapy	Proton therapy
Group R	CDF	+	-	-	-
	CDF	-	-	-	-
	AWD	+	+	-	-
	Dead	+	-	-	-
	Dead	+	-	-	-
	Dead	-	+	+	-
Group N	Dead	+	+	-	-
	Dead	+	+	-	-
	Dead	+	-	-	-
	Dead	-	-	-	-

In the R group, all six patients underwent tumor resection with spinal reconstruction and subsequently received various concomitant oncologic treatments. Chemotherapy was administered to four patients, radiation therapy to two patients, and carbon-ion radiotherapy to one patient. Among patients who received chemotherapy, final outcomes included CDF survival in one patient, survival with persistent disease in one patient, and death in two patients. Among patients who received radiation therapy, one patient was AWD at the final follow-up, and one patient died. The patient who underwent carbon-ion radiotherapy had died by the final follow-up. In contrast, among patients who did not receive postoperative oncologic therapy after resection, CDF survival was observed at the final follow-up.

In the N group, none of the patients underwent surgical treatment, and all were managed with nonsurgical oncologic therapies. Chemotherapy was administered to three patients, and radiation therapy was administered to two patients. All patients treated with chemotherapy died by the final follow-up, as did all patients who received radiation therapy. At the final follow-up, no patients in the non-resection group survived, regardless of the type or combination of nonsurgical therapies received (Table 2).

## Discussion

Summary of the findings

Primary spinal sarcomas represent a rare and heterogeneous group of malignant tumors, accounting for less than 5% of all primary musculoskeletal sarcomas [1]. Their unique anatomical constraints, including proximity to the spinal cord, nerve roots, major vessels, and critical stabilizing structures, make oncological control particularly challenging, and surgical decision-making is often complex. In the present study, patients who underwent resection and reconstruction tended to show more favorable oncological and functional outcomes than those who did not undergo resection; however, due to the limited number of cases, a statistically significant survival benefit could not be demonstrated. In contrast, postoperative functional outcomes, including preservation of ambulation, were consistently better among patients who underwent surgery.

Impact of surgical resection on oncological outcomes

Although the resection group demonstrated a higher five-year OS rate (60% vs. 0%), this difference was not statistically significant in the present series. This finding should be interpreted with caution, as the small cohort size substantially limited statistical power. Therefore, these findings should be interpreted as representing a trend rather than definitive evidence of survival benefit. Nevertheless, the observed survival trend was consistent with previous reports, emphasizing the importance of achieving adequate surgical margins in spinal sarcoma management. In primary chondrosarcoma of the spine, en bloc resection has been shown to substantially reduce local recurrence and distant metastasis, whereas inadequate margins are associated with recurrence rates approaching 100% [3-8].

Osteosarcoma of the spine remains a therapeutic challenge because complex spinal anatomy frequently precludes wide resection, and outcomes remain poor when adequate margins cannot be achieved [9-15]. Similarly, Ewing sarcoma arising in the spine has been associated with poorer outcomes than extremity lesions, although surgical resection may improve local control when feasible [16-20].

Challenges and limitations of surgical management

It is essential to acknowledge that patients selected for surgical treatment inherently represent a biologically and anatomically favorable subset. Tumors amenable to resection are typically smaller, more localized, and occur in patients with relatively preserved performance status. In contrast, extensive local invasion, poor general condition, or advanced disease often preclude surgical intervention. Moreover, surgical management of primary spinal sarcomas is associated with substantial perioperative risks, including massive blood loss, neurological morbidity, and mechanical complications related to reconstruction. These challenges underscore the need for treatment at specialized centers with experienced multidisciplinary teams. Therefore, although our findings support surgical resection as the preferred treatment strategy when feasible, surgical decision-making must be individualized and based on meticulous preoperative evaluation. This evaluation should comprehensively consider tumor location and extent, including the WBB and Enneking staging systems, degree of neurovascular involvement, feasibility of achieving acceptable surgical margins through en bloc excision, and patient-specific factors such as systemic condition, comorbidities, and anticipated functional goals.

Role of definitive radiotherapy in unresectable disease

Definitive radiotherapy is an important local treatment strategy for patients who are not candidates for surgical resection. Advances in particle therapy, including proton beam therapy and carbon-ion radiotherapy, have enabled the delivery of high-dose radiation with superior dose conformity while sparing surrounding critical structures such as the spinal cord. Recent consensus statements indicate that particle therapy allows dose escalation with acceptable toxicity in spinal tumors, particularly when surgical resection is not feasible [21,22]. Clinical series focusing on primary spinal and paraspinal sarcomas treated with carbon-ion radiotherapy have reported favorable local control in selected patients, although outcomes remain heterogeneous and highly dependent on tumor biology [23,24]. Furthermore, systematic reviews suggest that carbon-ion radiotherapy offers a potential local treatment option for unresectable bone sarcomas while emphasizing that durable disease control cannot be uniformly achieved without surgery [25]. Therefore, although particle therapy may provide meaningful local control and symptom palliation, it should be regarded as a complementary modality rather than a replacement for surgical resection, especially for anatomically challenging spinal tumors [26].

In the present study, the detailed patient-level data summarized in Table 2 further indicate that overall oncological outcomes were not clearly stratified according to the type or presence of concomitant oncological therapies. Patients who received chemotherapy, radiation therapy, or carbon-ion radiotherapy demonstrated heterogeneous clinical courses, including prolonged survival and early mortality.

In contrast, the most consistent difference in outcomes was observed between patients who underwent surgical resection with reconstruction and those who did not. Regardless of the specific combination of adjuvant or definitive nonsurgical treatments, patients in the non-resection group had poor survival, whereas a subset of patients in the resection group achieved prolonged disease control. Although the small sample size precluded formal statistical evaluation of treatment-specific effects, these observations suggest that surgical resectability may be an important contributing factor to clinical outcomes, potentially exerting a greater influence than the use or type of concomitant therapy in primary high-grade spinal sarcoma. As demonstrated in Table 2, individual patient outcomes varied considerably depending on tumor biology and treatment combinations, highlighting the heterogeneous nature of this disease.

Functional outcomes and the importance of mechanical stability

In patients with primary spinal sarcoma, preservation of postoperative function represents a major clinical challenge owing to tumor-related instability, neurological compromise, and the need for extensive local treatment. Surgical resection with spinal reconstruction provides mechanical stability, which is essential for maintaining ambulation, postural alignment, and independence in ADL. In contrast, nonsurgical management often fails to adequately control progressive spinal instability, deformity, and pain, potentially leading to deterioration of neurological function and loss of mobility over time.

In the present study, preservation of ambulation was markedly more frequent in patients who underwent resection and reconstruction than in those who did not. This finding suggests that mechanical stabilization achieved through surgical reconstruction plays a central role in functional recovery, independent of its impact on oncological outcomes. Maintenance of ambulation has been associated with improved quality of life and has been reported as an important prognostic factor for survival in patients with both primary and metastatic spinal tumors [27-29].

These observations suggest that the clinical value of surgical intervention for primary spinal sarcomas may not be fully captured by survival metrics alone. While OS remains an important outcome measure, functional outcomes, such as preservation of ambulation and independence in ADL, represent clinically meaningful endpoints that may better reflect patient benefit in this setting. Given the anatomical complexity of the spine and the functional consequences of tumor-related instability and neurological compromise, consideration of postoperative function is particularly relevant when evaluating treatment strategies for this rare disease.

Study limitations

This study has some inherent limitations. First, its retrospective design and small sample size reflected the extreme rarity of primary spinal sarcomas and limited the statistical power of the analysis. Importantly, potential confounding factors, including age differences, tumor characteristics, and treatment heterogeneity, were not adjusted for because of the small sample size and lack of multivariate analysis. Second, heterogeneity in histological subtypes and adjuvant treatments may have influenced both oncological and functional outcomes. Third, selection bias is unavoidable, as patients undergoing surgical resection tend to have more favorable anatomy and performance status. Accordingly, the present findings should be interpreted cautiously and regarded as hypothesis-generating.

While advances in particle therapy continue to expand treatment options for unresectable disease, these modalities currently complement, rather than replace, surgical resection. Therefore, optimal management requires individualized multidisciplinary decision-making that balances oncological control with functional preservation.

## Conclusions

In this retrospective series of primary high-grade spinal sarcomas, patients who underwent surgical resection with reconstruction tended to have more favorable oncological outcomes and better postoperative functional status than those treated without resection. Specifically, preservation of ambulation and independence in ADL were more frequently achieved in surgically treated patients, whereas outcomes remained poor in unresectable cases despite nonsurgical oncological therapies. Although a definitive survival benefit could not be demonstrated, surgical resection with reconstruction was associated with improved functional outcomes, particularly preservation of ambulation, suggesting a potential role in maintaining quality of life in patients with primary spinal sarcomas.
